# Adapting a Federal Disaster Medical Assistance Team to Operate During a Pandemic

**DOI:** 10.1017/dmp.2020.405

**Published:** 2020-10-23

**Authors:** Andrew L. Garrett

**Affiliations:** The George Washington University School of Medicine and Health Sciences, Washington, DC, USA

**Keywords:** Disaster Medicine, Field hospitals, Pandemics

## Abstract

After Hurricane Laura struck the southeast coast of Louisiana in August 2020, the National Disaster Medical System (NDMS), a component of the US Department of Health and Human Services, Office of the Assistant Secretary for Preparedness and Response, deployed several 35-person disaster medical assistance teams in response to requests for medical support at 3 hospital locations that had been severely damaged in the storm. This was the first natural disaster medical deployment for NDMS during the coronavirus disease (COVID-19) pandemic. This article describes the modifications to the standard operating procedures that were made at 1 site to reduce the risk of infection to our patients and NDMS responders, including changes to the physical layout of the tenting, and alterations to the triage and treatment process.

Hurricane Laura was a Category 4 storm on the Saffir-Simpson scale that made landfall near Cameron, Louisiana, in the early morning hours of August 27, 2020. The storm caused widespread major impact in the form of a storm surge, flash flooding, and heavy wind damage to homes and infrastructure in the southeast part of the state. Several hospitals in the southeastern part of the state sustained severe damage with impacts ranging from a reduction of inpatient and/or surgical services to full evacuation and temporary closure of the facility.

The State of Louisiana requested and received a major disaster declaration from the federal government to support their response to the incident, which placed the Federal Emergency Management Agency (FEMA) into the coordinating role for all federal assets. The US Department of Health and Human Services (HHS), the lead federal agency for public health and medical support (Emergency Support Function #8 under the National Response Framework), working with state and local health officials, determined that 35-person disaster medical assistance teams (DMATs) were needed to “decompress” several functional hospital emergency departments (EDs) that were attached to heavily impacted hospitals in the area.

Two of the DMATs deployed used a hybrid service model where they were integrated with the hospitals they were assisting, providing some tent-based medical care combined with National Disaster Medical System (NDMS) staff augmentation to the ED. The third DMAT, described in this manuscript, deployed an independent tent-based facility to assist a short-term, medical-surgical general hospital with approximately 150 beds in southeast Louisiana, a typical mission for NDMS. The hospital received significant damage and was without municipal power and water after the hurricane, forcing them to temporarily shut down their clinical laboratory, intensive care units, and surgical facilities and to greatly reduce the number of inpatients. Their ED remained open and experienced a surge in patients as one of the few remaining sources of emergency care in the community. Many of the other hospitals and most of the clinics and doctors’ offices remained closed during the week after the hurricane. Local emergency medical services (EMS) remained operational and were supplemented by additional ambulances from outside jurisdictions, increasing the demand on the ED. The DMAT was assigned its mission on post-storm day (PSD) 3, the team arrived at the end of PSD 4, and began 24/7 operations on PSD 5.

## Narrative

Upon receipt of the mission assignment and prior to departure on PSD 4, Assistant Secretary for Preparedness and Response (ASPR) and NDMS personnel convened a conference call with hospital administration to discuss the facility’s requirements and to agree upon the capabilities that the team could provide. An advance team from ASPR made a site visit on PSDs 3 and 4 to determine the best location to establish the DMAT base of operations (BoO) prior to the arrival of the full team the next day. Setting mutual expectations prior to the arrival of the DMAT was instrumental to the success of the mission.

The responding DMAT was a standard 35-person package that is composed of physicians, nurse practitioners, physician assistants, registered nurses, paramedics, pharmacists, a safety officer, and command and control staff. DMATs are comprised of intermittent federal employees of NDMS who are currently licensed in their home state at their designated provider level. While deployed, NDMS employees are authorized to deliver medical care at their licensed level in any jurisdiction of the United States without the need to apply for reciprocity, and they receive federal liability and workers compensation coverage. DMATs are not classified as World Health Organization Emergency Medical Teams (EMTs); however, they include some capabilities typical in Type-I fixed and Type-2 EMTs in that they provide 24-hour services, stabilizing outpatient medical care and emergency care of children and adults. They do not, however, provide surgical or specialty services, or inpatient care.^1^ Separate deployable NDMS assets are available that perform damage control surgery, fixed or aeromedical transportation of critical care patients, veterinary care, patient tracking, and assistance with mortuary operations.

Due to the ongoing pandemic, the DMAT modified its normal operating procedures to facilitate the social distancing of responders and patients, and to accommodate the need for a comprehensive personal protective equipment (PPE) posture. DMATs have embedded safety and supervisory medical officers to ensure the safe operations of the group. Additionally, NDMS operations in the field are overseen by a forward-deployed ASPR Incident Management Team, which includes certified safety officers and a chief medical officer who provides operational guidance as well. A collaborative management approach is used to ensure that the team’s operational goals can be safely achieved, despite the hazardous conditions that can exist in the post-disaster environment.

**Figure 1.**
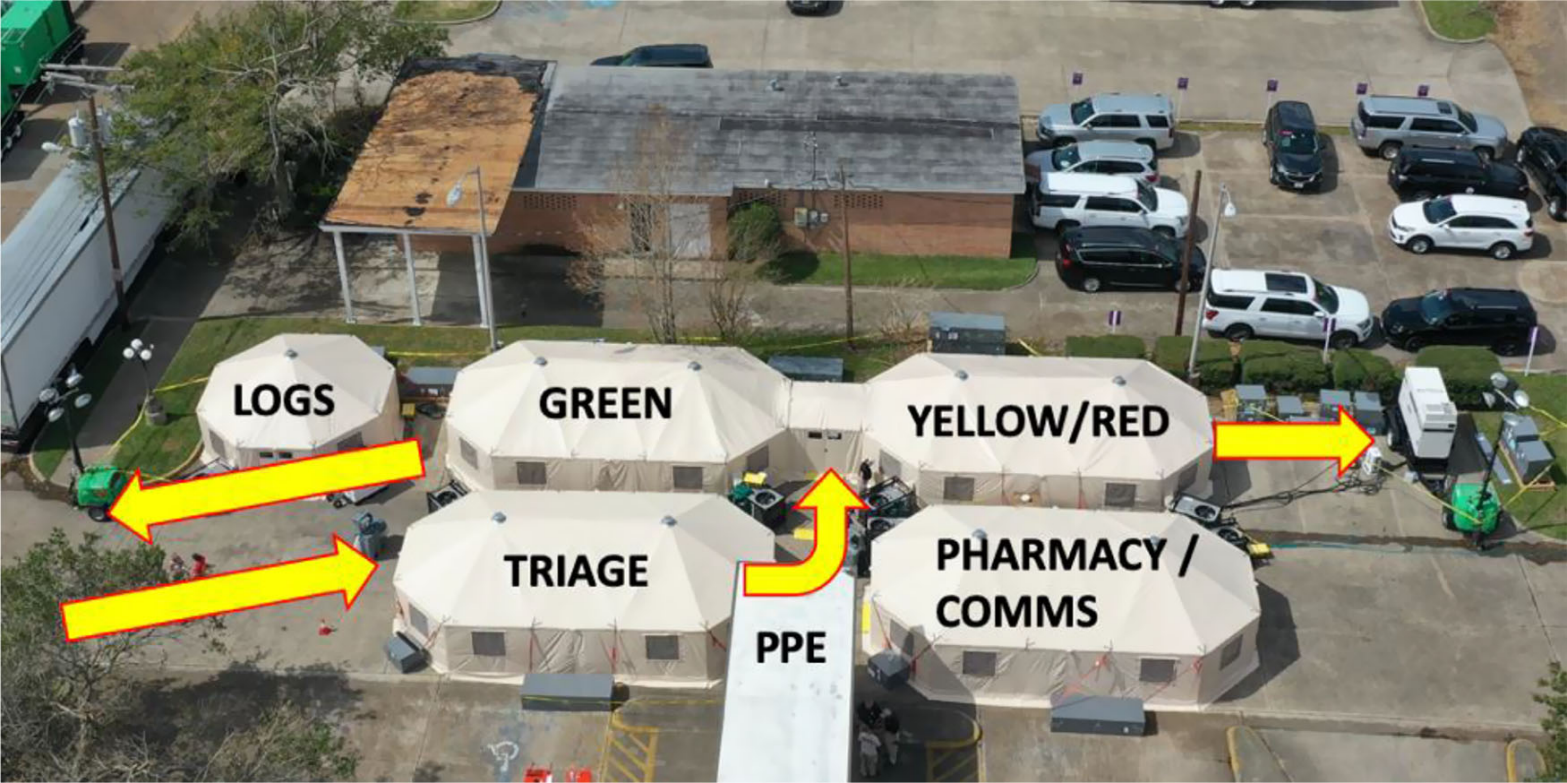
Overhead view of the DMAT base of operations for this deployment. Yellow arrows indicate the 1-way direction of patient movement through the facility. LOGS = logistics; COMMS = communications; PPE = personal protective equipment (donning station); and GREEN, YELLOW, and RED indicate the triage category for patients treated in the tent. Photo by Ryan Goodson.

Specific to operating with the ever-present coronavirus disease (COVID-19) threat, all NDMS employees were required to comply with the Centers for Disease Control and Prevention (CDC) recommendations for mask use, hand hygiene, and social distancing – whether on or off duty. Disposable surgical face masks were provided for employees in clinical areas unless they were providing direct patient care, at which point they were required to upgrade to an N-95 mask. All NDMS employees are fit tested annually for this equipment as a condition of deployment. Additionally, all NDMS providers who were in close proximity (6 feet or less) to a patient were required to wear eye protection or a face shield, and gloves. Disposable gowns were initially required for all patient contact, but this was changed to be at the discretion of the provider once daily laundry became available to wash team member uniforms after PSD 9. A large covered outdoor PPE donning station was established outside the triage and clinical tents (Figure 1), and handwashing stations, as well as hand sanitizing gel, were available at every entrance to a tent or NDMS-occupied area and throughout the treatment and administrative or support areas of the BoO. Stretchers were covered with disposable sheets that were changed after every patient. Wherever possible, disposable diagnostic equipment was used, and, where this was not possible, the equipment and patient areas were decontaminated using either a spray solution or pre-soaked wipes with a quaternary solution that was accepted by the Environmental Protection Agency for surface cleaning and sanitization. Sharps, as well as contaminated or medical waste generated by the DMAT, were red bagged and managed by a contracted certified waste management company.

With the concurrence of the hospital ED, NDMS co-located a nurse or paramedic with a portable radio with hospital staff at the ED triage point to determine whether patients would stay in the hospital (ED) or be moved to the DMAT. Each patient arriving by EMS or as a walk-in was assessed for signs and symptoms of COVID-19 based on CDC guidance along with the chief complaint for their visit, with the intent of keeping potentially infected patients isolated in the ED in order the reduce the risk to providers and other patients.^2^ Patients who would likely require ancillary services, such as imaging, or who were assessed as having an Emergency Severity Index of 1 or 2 were not normally treated in the DMAT tents.

**Figure 2.**
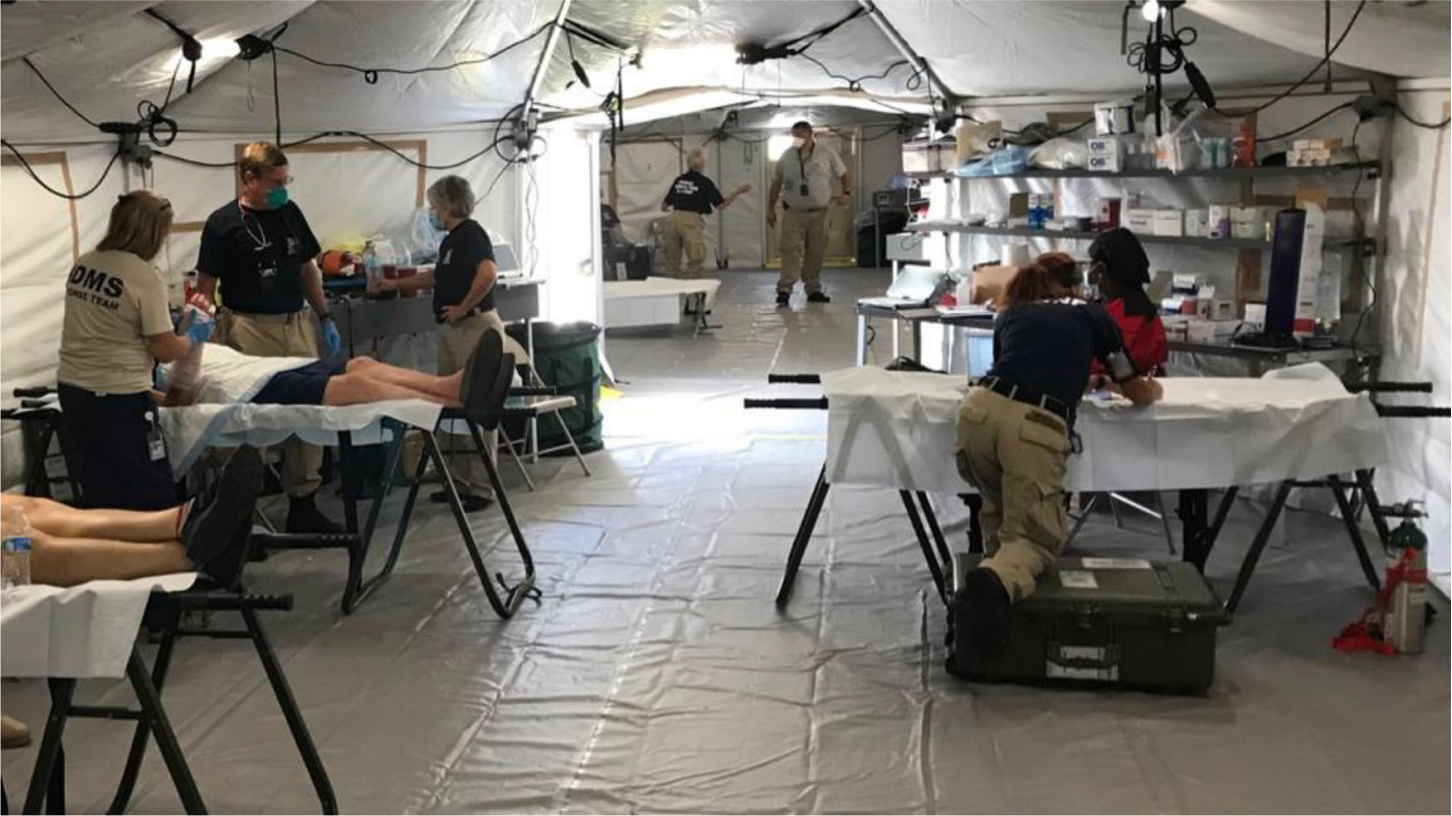
The spacing between recumbent patient care stations was increased on the Yellow/Red side of the treatment tent. Photo by Andrew Garrett.

To accommodate social distancing for patients, the DMAT increased its spacing between patient care stations and initiated 1-way flow for patients through the treatment tents, which were separated for minor (triage category *green*) patients and intermediate or emergent patients (triage categories *yellow* and *red*) (see Figure 1). A minimum of 6 feet or more was established between chairs or stretchers in the treatment tents, effectively reducing the capacity of the tents by at least 50% (Figure 2). Prior to the pandemic, chairs and stretchers were typically placed as close as possible to maximize capacity but still permit access to patients. Patient flow was controlled by staff direction and the use of yellow caution tape, orange cones, and appropriate signage. Patients were asked to wear surgical masks and to wash their hands prior to entering the medical treatment area. Airflow in the tents was increased through the use of additional air conditioning units, and high-efficiency particulate air (HEPA) filters were requested for the air handling unit returns. Additionally, contracted services were obtained to clean and sanitize the entire BoO several times daily using CDC-recommended guidance.

Responder meals and overnight billeting operations were modified as well. There were no multiple-serving packages permitted for snacks or meals, and social distancing was enforced during break times and in cohorted sleeping situations. Ventilation was increased in areas where staff congregated for break time and sleeping, and HEPA recirculating fans were provided by the hospital in the cohorted sleeping area. All NDMS employees were required to participate in daily monitoring for signs and symptoms of COVID-19 using a text-message-based system, which was overseen by health and safety personnel at NDMS headquarters. This supervised daily monitoring continued for 14 days after arriving home after deployment. No-contact thermometers were provided for staff to use at shift change to encourage self-monitoring.

## Discussion

With the screening of patients at hospital triage and disposition of higher risk patients to the ED facility, the DMAT was able to mitigate some of the risk to its providers and patients posed by COVID-19. If the mission requirements had included the treatment of known or higher risk COVID-19 patients, additional modifications to the above procedures would have been needed, such as the requirement that all providers wear gowns with all patients and the construction of partial barriers between patient care stations.

Overall, deploying a DMAT for a traditional disaster medical mission during a pandemic proved to be anything but routine. Close cohorting, sometimes to an extreme, has traditionally been the norm during field medical operations for both patients and on- and off-duty responders. NDMS had to re-evaluate every aspect of the deployment, including air and ground transportation, patient assessment and treatment, and responder feeding and billeting, to ensure that proper preventive actions and social distancing were continuously enforced. Compounding this challenge were the environmental conditions at the worksite, including extreme heat and humidity (daily heat indices were in excess of 106°F [41°C]), which made the routine use of PPE extremely difficult for personnel who needed to be working outside, especially during the construction of the BoO. Despite this, the team observed a high degree of compliance with preventive requirements and demonstrated that disaster field medical operations can be done with reasonable safety in the time of COVID-19 when appropriate modifications are undertaken.

## References

[1] World Health Organization Emergency Medical Teams Classification (2018, January 25). Retrieved September 1, 2020 from https://extranet.who.int/emt/emt-classification

[2] Centers for Disease Control and Prevention Symptoms of Coronavirus (2020, May 13). Retrieved September 18, 2020 from https://www.cdc.gov/coronavirus/2019-ncov/symptoms-testing/symptoms.html

